# Marked variation in heritability estimates of left ventricular mass depending on modality of measurement

**DOI:** 10.1038/s41598-019-49961-w

**Published:** 2019-09-19

**Authors:** Richard M. Nethononda, Kathryn A. McGurk, Polly Whitworth, Jane Francis, Chysovalanto Mamasoula, Heather J. Cordell, Stefan Neubauer, Bernard D. Keavney, Bongani M. Mayosi, Martin Farrall, Hugh Watkins

**Affiliations:** 1 Division of Cardiology, Chris Hani Baragwanath Hospital, Soweto and the University of Witwatersrand, Johannesburg, South Africa; 2 Division of Cardiovascular Sciences, Faculty of Biology, Medicine and Health, University of Manchester, Manchester, UK; 3 Division of Cardiovascular Medicine, Radcliffe Department of Medicine, University of Oxford, Oxford, UK; 4 Oxford Cardiovascular Clinical Research Facility (CCRF), John Radcliffe Hospital, Oxford, UK; 5 Oxford Centre for Clinical Magnetic Resonance Research (OCMR), John Radcliffe Hospital, Oxford, UK; 6 Institute of Genetic Medicine, Newcastle University, Newcastle upon Tyne, UK; 7 Department of Medicine, University of Cape Town, Cape Town, South Africa; 8 Wellcome Centre for Human Genetics, University of Oxford, Oxford, UK

**Keywords:** Heritable quantitative trait, Cardiovascular genetics

## Abstract

Left ventricular (LV) hypertrophy is a strong risk factor for heart failure and cardiovascular death. ECG measures of LV mass are estimated as heritable in twin and family-based analyses and heritability estimates of LV mass measured by echocardiography are lower. We hypothesised that CMR-derived measurements, being more precise than echocardiographic measurements, would advance our understanding of heritable LV traits. We phenotyped 116 British families (427 individuals) by CMR and ECG, and undertook heritability analyses using variance-components (QTDT) and GWAS SNP-based (GCTA-GREML) methods. ECG-based traits such as LV mass and Sokolow-Lyon duration showed substantial estimates of heritability (60%), whereas CMR-derived LV mass was only modestly heritable (20%). However, the ECG LV mass was positively correlated with the lateral diameter of the chest (rho = 0.67), and adjustment for this attenuated the heritability estimate (42%). Finally, CMR-derived right ventricular mass showed considerable heritability (44%). Heritability estimates of LV phenotypes show substantial variation depending on the modality of measurement, being greater when measured by ECG than CMR. This may reflect the differences between electrophysiological as opposed to anatomical hypertrophy. However, ECG LV hypertrophy traits are likely to be influenced by genetic association with anthropometric measures, inflating their overall measured heritability.

## Introduction

Left ventricular hypertrophy (LVH) and mass (LVM), whether measured with ECG or echocardiography, is a strong predictor of cardiovascular morbidity and mortality^1^. However, prognostic information provided by ECG is independent but complementary to that provided by echocardiography^2^. An explanation for this discrepancy may derive from the fact that the ECG LVM reflects electrical activities within myocardium, a property of the mass and electrophysiological state of cardiomyocytes, and electrical impedance between the heart and the skin; whereas echocardiographic LVM reflects a combination of myocardial and interstitial mass. We have previously collected ECG and echocardiographic data from a series of white British families ascertained through a hypertensive proband and found that ECG-derived mass was more heritable than Echo-derived LVM with the Sokolow-Lyon voltage index showing the highest heritability^3^. One possible explanation for this finding was that high variability of echocardiography in quantifying LVM and wall thickness could mask the heritability of these phenotypic traits compared to ECG-based measurements. Cardiac magnetic resonance (CMR) has been shown to be highly accurate and reproducible in quantification of left and right ventricular volumes, mass and ejection fraction^4,5^. These observations led to the hypothesis that more accurate quantification of LV phenotypes by CMR could provide better assessment of LV heritability estimates compared to those obtained by echocardiography. Thus CMR might resolve heritable LVH traits potentially strongly linked to cardiovascular mortality and morbidity that are optimally tractable to genetic analysis.

We therefore re-phenotyped the British family panel using CMR to measure left and right ventricular volumes and mass. We also conducted genome-wide genotyping enabling estimation of SNP-based heritability and conduct of a GWAS for LVM. These data allow a direct comparison of heritability estimates of ECG-derived indices of LVH with those obtained by CMR.

## Results

### Population characteristics

Baseline characteristics of the study population are shown in Table 1. The mean age was 59.7 ± 13 years and 52.5% were females. In general the study population was overweight with a BMI of 28.1 ± 4.8. Office systolic and diastolic blood pressures (BP) were 140.5 ± 18.2 and 81.5 ± 10.3 mmHg, whereas the mean 24 h systolic and diastolic BP were 130.8 ± 13.3 and 77.3 ± 7.9 mmHg, respectively. Both these blood pressures were direct measured BP without adjustment. Based on the 2018 ESH/ESC guidelines on hypertension^6^ both the office systolic blood pressure (SBP) and the 24-h ambulatory SBP were in the hypertension range.

**Table 1:** Baseline characteristics (n = 427).

Age, yrs	59.7 ± 13
Females, n (%)	228 (52.5)
Weight (kg)	79.3 (68.7–89.2)
Height (m)	1.69 ± 0.09
BMI (m/kg^2^)	28.1 ± 4.8
BSA (m^2^)	1.53 ± 0.24
SBP (mmHg)	140.5 ± 18.2
DBP (mmHg)	81.5 ± 10.3

Data presented as mean ± SD or as median (IQR) or as numbers of participants (%); BMI, body mass index; BSA, body surface areas; S/DBP, systolic/diastolic office blood pressure.

### Measures of left ventricular phenotypes

Table 2 lists descriptive statistics for the ECG measures of LVH and LVM. Twenty-two individuals could not be phenotyped due to bundle branch block (n = 12), atrial fibrillation (n = 5), or presence of permanent pacemaker (n = 5). Both the Sokolow-Lyon voltage (21.1 ± 6.5 mV in males; 19.0 ± 5.3 mV in females) and the Cornell voltage (12.9 ± 4.8 mV in males; 11.7 ± 4.8 mV in females) were below the respective cut-off values (Sokolow-Lyon of 35 mV for both genders; and Cornel voltage of 28 mV in males and 20 mV in Females) for the diagnosis of LVH^7^. The duration products of these indices were similarly below their respective LVH thresholds of 2840 mV.ms (Sokolow-Lyon duration) and 2440 mV.ms (Cornell duration). Finally, the calculated ECG LV masses [mean of 130.2 g (114.9 g –143.5 g)] were also in the normal range.

**Table 2:** ECG indices of left ventricular hypertrophy.

	Male (n = 195)	Female (n = 210)
Sokolow-Lyon (mV)	21.1 ± 6.5	19.0 ± 5.3
Sokolow-Lyon product (mV/s)	1860.8 ± 546.2	1626.5 ± 466.8
Cornell (mV)	12.9 ± 4.8	11.7 ± 4.8
Cornell Product (mV/s)	1209.9 ± 500	1015.5 ± 456.6
12 lead sum QRS voltage (mV)	129.3 ± 23.1	116 ± 22.3
12 lead sum QRS product (mV/s)	11932.6 ± 2844.8	10216 ± 2482.9
LVM (g)	143.9 ± 17.7	119.6 ± 17.1

Data presented as mean ± SD; LVM, left ventricular mass. n = maximum number of phenotyped individuals available for heritability analysis.

Table 3 lists descriptive statistics for CMR LV volumes and mass and derivative indices (by body surface area). Twenty-two subjects were excluded from this analysis due to claustrophobia (n = 8), presence of a pacemaker (n = 5), obesity (n = 2), unwillingness to undergo examination (n = 5), aneurysm clips (n = 1), or backache (n = 1). In general left and right ventricular volumes indices were in the same ranges as those previously reported^8^. Males had larger ventricular volumes than females. LVM indices were generally higher than those previously reported, in keeping with the ascertainment strategy for this cohort.

**Table 3:** CMR measures of left ventricular hypertrophy.

	Male (n = 195)	Female (n = 210)
LVM (g)	149.2 ± 31.5	106.1 ± 23.2
LVM index (g/m^2^)	73.7 ± 13.5	59.3 ± 10.9
LV end-diastolic volume (mL)	149.4 ± 29.3	121.1 ± 25.1
LV end-diastolic volume index (mL/m^2^)	74.0 ± 13.9	67.7 ± 11.8
LV end-systolic volume (mL)	44.2 ± 12.3	33.9 ± 11.4
LV end-systolic volume index (mL/m^2^)	21.9 ± 6.0	19.0 ± 6.0
LVH (%)	22.6	22.1.1

Data presented as mean ± SD. LVM, left ventricular mass; LVH, left ventricular hypertrophy. n = maximum number of individuals available for heritability analysis.

### Estimates of heritability

The numbers of families and individuals with phenotype data available for heritability analysis are shown in supplementary Table 1. Families with a solitary phenotyped member can contribute to the GCTA-GREML heritability estimates (h^2^_IBS>0.05_) if their genotype data indicate cryptic relationships, but not the QTDT variance-component estimates (h^2^_QTDT_), which rely on self-reported relationships and family structures. Table 4 and Supplementary Tables 3–6 show heritability estimates for ECG and CMR LV hypertrophy phenotypes after adjustment for significant (*P*-value < 0.01) sources of covariation. Consistent with previously published data from this cohort^3^, ECG Sokolow-Lyon voltage showed moderate heritability in both the variance-components (QTDT) and IBS threshold (GCTA-GREML) analyses (h^2^_QTDT_, 35 ± 9%; h^2^_IBS>0.05_, 40 ± 11%). These estimates increased when the phenotype was expressed as a product with the QRS duration, the Sokolow-Lyon duration (h^2^_QTDT_, 57 ± 9%; h^2^_IBS>0.05_, 60 ± 10%); on paired statistics this was not statistically different (*P-value* = 0.7) from the heritability estimate of ECG LVM (h^2^_QTDT_, 62 ± 9%; h^2^_IBS>0.05_, 60 ± 10%).

**Table 4:** Heritability estimates for CMR and ECG LV phenotypes.

	phenotype	number of phenotyped individuals	QTDT			GCTA-GREML			
			h^2^_QTDT_ (%)	SE (%)	*P*-value	no. genotyped	h^2^_IBS>0.05_ (%)	SE (%)	*P*-value
CMR	LVM	397	17	9	0.0469	362	27	12	0.0230
	LVM index	397	17	8	0.0402	362	27	12	0.0196
	LV end-diastolic volume	405	41	10	3.5E-05	369	47	12	7.2E-05
	LV end-diastolic volume index	405	42	10	1.7E-05	369	47	12	5.9E-05
	LV end-systolic volume*	397	32	10	0.0012	362	33	12	0.0049
	LV end-systolic volume index*	397	35	10	0.0004	362	36	12	0.0019
ECG	Cornell voltage	404	25	9	7.4E-03	364	21	11	0.0541
	Cornell duration	404	29	9	8.8E-04	364	26	11	0.0129
	LVM^†^	404	62	9	3.5E-11	364	60	10	8.5E-09
	LVM^‡^	362	42	16	0.0087	330	43	11	2.2E-06
	Sokolow-Lyon voltage	404	35	9	0.0001	364	40	11	0.0003
	Sokolw-Lyon duration	403	57	9	9.7E-10	363	60	10	8.2E-0.9

LV, left ventricular. ^†^LVM adjusted for significant (*P*-value < 0.01) covariation in height, treatment-adjusted SBP and DBP and sex, ^‡^LVM adjusted for significant (*P*-value < 0.01) covariation in CMR chest lateral diameter, height and treatment-adjusted SBP. *GCTA-GREML models were fitted without “–no-constrain” flag.

**Figure 1:**
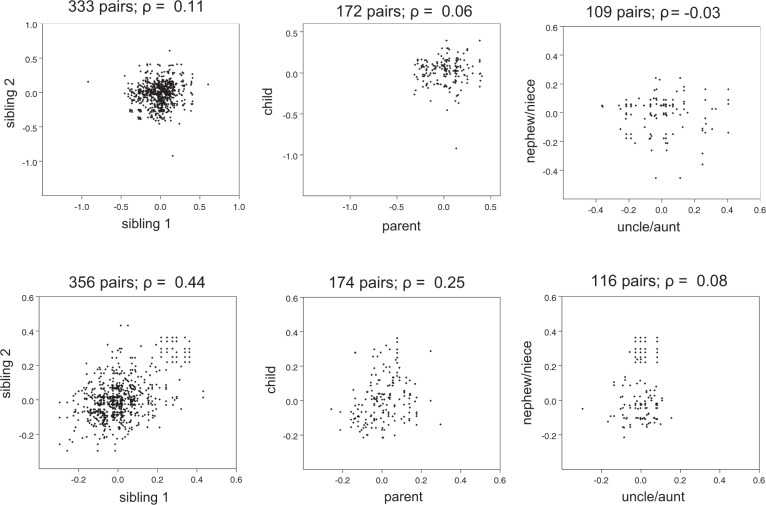
Scatter plots of left ventricular mass measures for pairs of relatives. The upper 3 plots show phenotype residuals following adjustment for covariation for cardiac MR left ventricular mass for sibling, parent-child or uncle/aunt-nephew/niece pairs; the lower plots show the corresponding residuals for the ECG left ventricular mass measurements. The number of phenotyped relative-pairs and the Pearson correlation coefficient (r) are shown above each plot.

**Figure 2:**
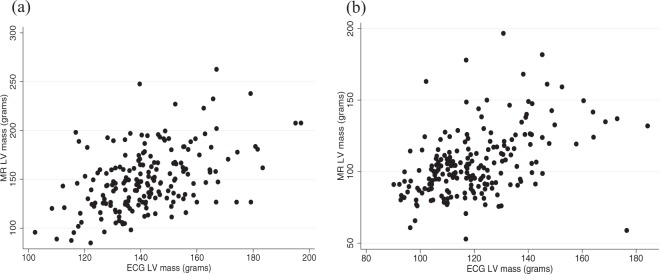
Correlations of cardiac MR and ECG measures of left ventricular (LV) mass in males (**a**) and females (**b**). Masses are unadjusted for potential sources of covariation.

CMR LV phenotypes all showed nominally significant evidence of heritability (*P*-value < 0.05), with LV end diastolic volume index showing the strongest evidence (h^2^_QTDT_, 42 ± 10%; h^2^_IBS>0.05_, 47 ± 12%). The CMR LVM showed weaker heritability compared to the ECG LVM (h^2^_QTDT_, 17 ± 9%; h^2^_IBS>0.05_, 27 ± 12%), although the difference was insignificant (heterogeneity *P-value* = 0.5). Figure 1 shows the intra-pair correlations for 1^st^ and 2^nd^ degree relative pairs that underpin the ECG and CMR LVM heritability estimates. Figure 2 shows correlation between ECG and CMR LVM in males (a) (r = 0.47) and females (b) (r = 0.46), respectively. On Fisher’s z-transformation there was no significant difference in these correlations (*P-value* = 0.9). For the combined group the rho is 0.65. Supplementary Figure 1 also shows correlation between ECG LVM adjusted for height and CMR LVM in males (a) (r = 0.41) and females (b) (r = 0.37), respectively (Fisher z-transformation *P-value* = 0.65).

Supplementary Tables 4 and 6 show heritability estimates for CMR right ventricular (RV) and chest dimensional phenotypes. Several of the RV traits show evidence of heritability, the strongest being RV mass and RV mass index (h^2^_QTDT_, 44 ± 9%; h^2^_IBS>0.05_, 45 ± 12%). Amongst the chest size phenotypes, chest lateral diameter showed the strongest evidence of heritability (h^2^_QTDT_, 60 ± 11%; h^2^_IBS>0.05_, 60 ± 11%). We found that chest lateral diameter showed a strong correlation with ECG LVM in our data (rho = 0.67, *P*-value = 4 × 10^−50^) raising the hypothesis that the ECG LVM heritability estimate is inflated due to confounding with chest size. We therefore re-examined the ECG LVM phenotype allowing for significant (*P*-value < 0.01) covariation in CMR chest lateral diameter, height and treatment-adjusted SBP, the resulting ECG LVM heritability estimate was duly attenuated (h^2^_QTDT_, 42 ± 16%; h^2^_IBS>0.05_, 43 ± 11%, Table 4).

**Figure 3:**
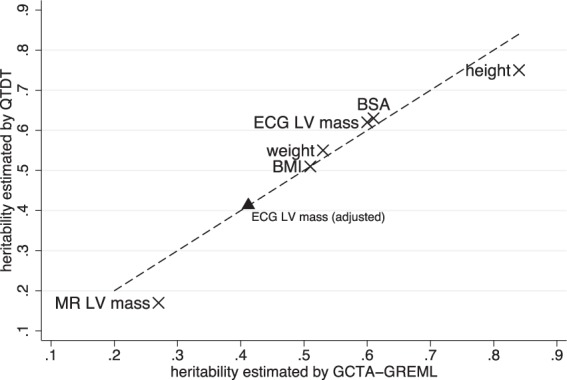
Comparison of heritability estimates from variance-component (QTDT) and IBS threshold (GCTA-GREML) analysis. MR LV mass, cardiac MRI LVM; ECG LV mass, electrocardiographic LVM; BMI, body mass index; BSA, body surface area. The line-of-identity (i.e. Y = X) is indicated by a dashed line. The QTDT heritability estimate for ECG LVM after adjustment for chest size is shown by a solid triangle.

Supplementary Tables 2 and 6 report heritability estimates for several anthropometric phenotypes. Of note is the high heritability estimate for height (h^2^_QTDT_, 75 ± 8%; h^2^_IBS>0.05_, 84 ± 8%), a quantitative inherited trait proven highly tractable to GWAS^9^. Figure 3 illustrates the overall consistency of the variance-components (QTDT) and IBS threshold (GCTA-GREML) heritability estimates for the ECG and CMR LVM, and the anthropometric traits. As expected with this sample number, the traits’ associations did not reach GWAS significance (*P-value* < 5 × 10^−8^).

## Discussion

We have revisited a family study in which we previously found that electrocardiographic parameters for LVH showed greater heritability than echocardiographic LVH measures^3^. We were uncertain as to whether those findings simply reflected the imprecision of echocardiography in the quantification of anatomic LVM or were an indication that the biological foundations of electrocardiographic measures of hypertrophy were inherited differently from those of anatomic hypertrophy. In addition, there was also a possibility that the higher heritability of ECG traits, particularly the Sokolow-Lyon voltage, which uses chest lead voltage, indirectly reflected the heritability of chest dimensions.

We therefore undertook to accurately assess the heritability of left ventricular phenotypes using CMR^4,5^. We also took advantage of the ability of the CMR to quantify right ventricular parameters to assess their heritability^4^. Furthermore, we used CMR to measure chest dimensions more precisely and assess their heritability relative to that of ECG indices of LVH.

We found very similar heritability estimates for three ECG phenotypes; Cornell voltage, Cornell duration, and Sokolow-Lyon voltage, to those reported in our previous study^3^. The ECG LVM heritability was though substantially larger (h^2^ ~ 0.6) than that reported (h^2^ ~ 0.18) in our previous study. Importantly, adjustment for co-variation in chest lateral dimension and height attenuated the ECG LVM heritability estimate (h^2^ ~ 0.42), suggesting that genetic influences on the highly heritable anthropometric indices that confound ECG LVM estimates, as well as cardiac electrophysiological and anatomical factors, contribute to the high heritability. As ECG voltages are influenced by potential confounding factors that could inflate heritability, we adjusted ECG LVM for factors which showed association with ECG voltages. Blood pressure affects LVM, therefore treatment-adjusted SBP was included in the adjusted model. Surface ECG voltages incorporate not only the electrical signal from the myocardial mass, but also factors related to the length and constitution of the tissue path through which the signal travels. To account for this, the ECG voltages were adjusted for chest lateral diameter and height. The uncorrected values can be interpreted as the heritability of ECG LVM due not only to genes influencing myocardial mass itself, but also genes affecting blood pressure, body stature, and habitus. The corrected values provide an estimate of the heritability due to genes acting chiefly or solely on myocardial mass.

The CMR LVM heritability estimates (h^2^_QTDT_ = 17%; h^2^_IBS>0.05_ = 27%) were comparable with those previously derived by echocardiography (h^2^ ~ 0.23). In contrast, RV mass showed more marked heritability (h^2^ ~ 0.44).

A key finding of the current study is that despite precise measurement of anatomic LVH using CMR, heritability estimates were broadly unchanged from those we previously reported in the same cohort using echocardiography^3^. In order to put our heritability estimates into context, we also estimated heritability for anthropometric traits and found, as expected, high estimates for phenotypes such as height, BSA, chest lateral diameter, weight, BMI, and chest volumes in descending order ranging from 84% to 40%. The contribution of these inflates the measured heritability of ECG measures of LVH, because of the effects of body habitus on the position of the heart and hence the electrocardiographic axes.

Several studies in different populations including the current cohort^3^ have reported heritability estimates for echocardiographic LVM ranging between 15 to 72%, with twin and sibling studies reporting higher estimates^10–13^. None of the studies examined heritability estimates for RV mass presumably because of the inherent difficulties in quantifying the right ventricle with echocardiography. We are aware of only one study that has reported heritability estimates for CMR LVM and papillary muscle mass in twin pairs, which found heritability estimates of up to 84% for LVM, 76% for end-diastolic volumes, and 27% for end-systolic volumes^14^. Therefore our study is the first to report on heritability estimates for CMR LVM and volumes in a cohort of nuclear and extended families. We have also taken advantage of GWAS SNP information to confirm the heritability estimates using a statistical method that is unbiased by gene-gene interactions and shared environmental factors^15^. We further exploited the precision of CMR to quantify RV phenotypes to report for the first time heritability estimates for RV mass and volumes. To our surprise we found that the RV mass was more heritable than the LVM. The RV has a different embryological origin from the LV and both before and after birth it is exposed to different haemodynamic loads and stresses. Our findings suggest that genetic influence plays a larger role in determining RV mass than on the LVM. However, we are uncertain as to the prognostic implications of a higher RV mass.

The recognition that ECG measures of myocardial mass are heritable has motivated researchers to apply GWAS methods to test the “common disease/common variant” hypothesis and scan for underlying quantitative trait loci (QTL). A recent large-scale meta-analysis of QRS traits identified several dozen such QTL, each one of which had a small influence on these heritable traits^16^. Collectively the variants explained a small proportion of the total phenotype variation; 2.7% for Sokolow-Lyon voltage and 5.0% for QRS duration, so a considerable portion of the anticipated heritability remains to be mapped (i.e. the “missing heritability”)^17^. Our findings suggest that genetic analyses of ECG LVM related phenotypes could be complicated by unrelated effects on chest size and other anthropometric measures, or pleiotropic effects on both cardiac biology and body anatomy, or effects on cardiac biology unrelated to hypertrophy per se, such as conduction influences on QRS duration. Therefore, optimally precise and interpretable analyses will require CMR as well as ECG data to identify specific LVH QTL effects.

## Methods

### Family ascertainment

Between 1993 and 1997, we recruited 248 families with 1425 members from Oxford for quantitative genetic study of hypertension and other cardiovascular risk factors^3^. Families were ascertained through a hypertensive proband (through a hospital hypertension service or via their family physicians) defined as daytime ambulatory blood pressure of >140/90 mmHg before the age of 65 years, a level which corresponds to the upper 5% of the distribution in Caucasian populations. This cohort comprises a panel of large extended white British families with adequate power to detect small genetic influence on quantitative traits^18^. For the current study, a power calculation was performed to establish the minimum sample size adequate to retain power for detection of small genetic influences on LVM and to compare heritability of LVM defined by CMR vs. ECG (see Statistical Analysis, below). The Central Oxford Research Ethics Committee approved the study, and all subjects gave written informed consent. The investigation conforms to the principles outlined in the Declaration of Helsinki.

### Phenotyping

Participants were phenotyped at different time-points. In the 1^st^ stage (1993–1997) we obtained demographic and anthropometric data, 24 h ambulatory blood pressure monitoring (ABPM), and blood samples for genetic analysis. In the 2^nd^ stage (1999–2000), 955 subjects from 229 families were recalled for phenotyping with electrocardiography, echocardiography, carotid B-mode ultrasound (CIMT), assessment of baroreceptor sensitivity, bioimpedance, and 24 h hour urine collection for steroid analysis; these data were used in our previous LVH heritability analysis^3^. In the 3^rd^ stage (2006–2008), 427 individuals from 116 families were recalled for CMR phenotyping. We also obtained ECG and contemporaneous 24 h ABPM, height, and weight measurements.

### Electrocardiography

12-lead ECG was acquired from a supine position using the atria 6100 system (cardioscience, UK) recorded at 25 mm/s and 1 mV/cm without filter. ECGs were blindly analysed by MRN. Leads amplitudes [millivolts (mV)] were measured from an average of three consecutive complexes. QRS amplitudes were measured from the peak of the R wave to the nadir of either the Q wave or the S wave, according to the method of Siegel and Roberts^19^. R-wave amplitudes were measured to the nearest 0.05 mV and QRS duration to the nearest 40 milliseconds (msec) from the beginning of the Q wave to the end of the S wave. The Sokolow-Lyon index (SL) was SV1 + RV5^20^. Cornell index (mV) was RaVL + SV3 in men and RaVL + SV3 + 0.6 mV in women as described^21^. The product (mV.ms) of these 2 indices was obtained by multiplying each one by the QRS duration in milliseconds. ECG LVM was calculated as 0·026 (RaVL + SV3) + 1·25 × weight + 34·4 for men; and 0·020 (RaVL + SV3) + 1·12 × weight + 36·2 for women^22^.

### CMR image acquisition

CMR was performed on a 1.5 Tesla scanner (Sonata; Siemens Medical Solutions, Erlangen, Germany) with a dedicated six-channel phased array surface coil as described by our group, among others^23^. Imaging was performed during end-expiration breath-hold. Scout images in axial, sagittal, and coronal planes were used to localise cardiac position and to plot double oblique, orthogonal, short and long axis imaging planes of the left ventricle. Segmented steady-state free precession (SSFP) cine imaging was performed for assessment of LV and RV volumes, mass and ejection fraction. Typical imaging parameters were as follows: repetition time (TR) 3.5 msec, echo time (TE) 1.6 msec, flip angle 60°, temporal resolution 35–40 msec, in-plane spatial resolution 1.9 mm × 1.4 mm. Images were acquired with a slice thickness of 7.0 mm and an interslice gap of 3.0 mm. The result was a horizontal long axis (HLA) image, a vertical long axis (VLA) image, and a left ventricular outflow tract view (LVOT). Using the HLA and VLA views obtained, we obtained a SA stack beginning from the atrioventricular groove and covering the entire left and right ventricle. CMR images were analysed using Argus software (version 2002B; Siemens Medical Solutions, Erlangen, Germany). Blinded manual tracing of the epicardial and endocardial borders of successive short-axis slices was performed (by MRN) in the standard way at end-diastole (phase with the image with the largest LV and RV cavity) and end-systole (phase with the image with the smallest LV and RV cavity). Epicardial and endocardial borders were traced on the end-diastolic frame, with only an endocardial border traced on the end-systolic frame. The basal slice was selected for the left ventricle when at least 50% of the blood volume was surrounded by myocardium in both end-diastole and end-systole. The apical slice was defined as the final slice showing intracavity blood pool at both end-diastole and end-systole. Papillary muscles were included in the mass and excluded from volume calculations. From these data, LVM, ejection fraction, and end-diastolic and end-systolic volumes were calculated. Myocardial mass was determined as tissue volume × 1.05 g/cm^3^ (specific density of myocardium)^24^. The LVM and volumes were indexed to body surface area. Black blood axial images were used to measure the antero-posterior diameter, defined by a vertical line from the anterior surface of the vertebral body to the mid-point of the sternum as bony landmarks. The line was extended anteriorly and posteriorly to the surface of the overlying skin, as well as the chest lateral diameter defined as the longest distance from the left to the right side of the chest, roughly mid-axillary from each side. The chest volume was derived from scout coronal images and was calculated using the formula for calculating the volume of a cylindrical body, i.e. πr^2^h, where r is half the lateral chest diameter and h is the height of the chest measured from the base (centre of the diaphragm) to the centre of the thoracic inlet.

### Genome-wide genotyping

DNA was extracted from whole blood by standard methods and quantified by a proprietary PicoGreen assay. Genotyping was performed using the Illumina 660W-Quad chip including 557,124 SNPs. Routine QC was undertaken using PLINK (version 1.9)^25^, to exclude SNPs with low call rates (<95%(-–geno 0.05)), individuals with low genotype rates (<95%(-–mind 0.05)), and SNPs with low minor allele frequency (<1% (-–maf 0.01)). Individuals with outlying heterozygosity were removed, as were SNPs that failed checks of Hardy-Weinberg Equilibrium (*P-value* < 1 × 10^−8^). Population stratification checks via principal components analysis with 1,000 Genome Projects individuals confirmed all participants were of European origin. Following QC, 503,855 autosomal SNPs were available for GCTA-GREML heritability analysis and family-based GWAS analysis.

### Statistical analysis

#### Covariate adjustment

Linear regression modelling for covariate adjustment and the subsequent heritability analysis assume that the error distributions have an approximate normal density, we therefore used the *boxcox* procedure in the Stata**™** (version 10.1) statistical analysis package to find the maximum likelihood parameters of the *boxcox* transform, and then transform the phenotype values accordingly (Supplementary Table 2). Important (*P-value* < 0.01) sources of covariation such as age, gender, height, weight, body surface area (BSA), body mass index (BMI), SBP or DBP were identified via multiple linear regression by applying a forward-backwards stepwise procedure in Stata**™** (version 10.1). SBP and DBP values were pre-adjusted for anti-hypertensive therapy by increasing SBP by 15 mm Hg and DBP by 10 mmHg^26^. Following covariate analysis, residual phenotype values were calculated and used in the subsequent heritability analyses.

#### Heritability and GWAS analyses

A variance-components heritability analysis was undertaken with the QTDT software^27^. Two statistical models, a general model that jointly estimated polygenic and random individual-specific effects was compared in a likelihood ratio test (LRT) with a nested random individual-specific effects-only model; standard errors (SE) of the resulting narrow-sense (additive effects-only) heritability estimates (h^2^_QTDT_) were calculated by approximating the LRT as a Wald test. Our primary aim was to measure the heritability of left and right ventricular mass and volume as measured by CMR to compare with heritable ECG phenotypes. We anticipated that these would lie in the range 25–50%. Exploratory power calculations, using simulation techniques to model polygenic inheritance in the family-structures suggested that 427 participants from 116 families would be sufficient to provide usefully precise estimates with a predicted sampling standard error of heritability estimate equal to 7.5%.

Genome-wide association (GWAS) data has been used to assess the overall heritability of phenotypes by fitting linear mixed-effects association models^28^. These estimates are complementary to those obtained from twin or familial correlation data analysed using variance-components models with the advantage that they are subject to less bias by epistasis (gene-gene interactions) or shared environmental factors. Yang’s method^28^ is however, itself biased when applied to mixtures of closely as well as distantly related individuals, an important consideration for the analysis of the extended families studied herein^15^. We therefore applied a single nucleotide polymorphism (SNP) driven, identity-by-state (IBS) threshold analytic approach to calculate h^2^_IBS>0.05_ heritability estimates; these estimates have been shown to accurately approximate fine-scale identity-by-descent (IBD) heritability estimates to provide unbiased GWAS-based estimates of narrow-sense heritability^15^. The h^2^_IBS>0.05_ calculations were performed using revised GCTA-GREML software (version 1.26.0)^29^. Linear mixed modelling approaches account for population substructure and relatedness in genome-wide association studies. Family-based genome-wide association analyses were undertaken for each trait using FAST-LMM (version v2.07.20140304) using the–ML command for maximum likelihood parameter learning^30^.

## Supplementary information

Supplementary Information

## Data Availability

GWAS summary statistics, relevant phenotype data, and analysis protocols are available from the authors on request.
