# Supplementary material for: Marked variation in heritability estimates of left ventricular mass depending on modality of measurement
Source: Sci Rep. 2019 Sep 19;9:13556. doi: 10.1038/s41598-019-49961-w (PMC6753112; doi:10.1038/s41598-019-49961-w)
Supplement: Supplementary file 1 — Supplementary Information [file 41598_2019_49961_MOESM1_ESM.pdf]

SUPPLEMENTAL MATERIALS

Marked variation in heritability estimates of left ventricular mass depending on modality of measurement

Richard M Nethononda MBchB, DPhil, FESC[1]; Kathryn A. McGurk BA[2]; Polly Whitworth SGN[3,4]; Jane Francis DCR, DNM[3,5]; Chysovalanto Mamasoula MSc[6]; Heather J Cordell DPhil[6]; Stefan Neubauer MD, FMEDSCI, FRCP, FACC[3,5,8]; Bernard Keavney DM FRCP[2]; Bongani Mayosi DPhil[7,9]; Martin Farrall FRCPath[3,8]; and Hugh Watkins MD PhD[3,8] *

Authors Affiliation:

[1]Division of Cardiology, Chris Hani Baragwanath Hospital, Soweto and the University of Witwatersrand, Johannesburg, South Africa; [2]Division of Cardiovascular Sciences, University of Manchester, Manchester, UK; [3]Division of Cardiovascular Medicine, Radcliffe Department of Medicine, University of Oxford, Oxford, UK; [4]Oxford Cardiovascular Clinical Research Facility (CCRF), John Radcliffe Hospital, Oxford, UK; [5]Oxford Centre for Clinical Magnetic Resonance Research (OCMR), John Radcliffe Hospital, Oxford, UK; [6]Institute of Genetic Medicine, Newcastle University, Newcastle upon Tyne, UK; [7]Department of Medicine, University of Cape Town, Cape Town, South Africa; [8]Wellcome Centre for Human Genetics, University of Oxford, Oxford; [9]Deceased.

Table 1. Number of families and phenotyped individuals included in heritability analysis

	ECG study		CMR LV study	
no. individuals per family	no. families	no. individuals	no. families	no. individuals
1	21	21	20	20
2	24	48	25	50
3	21	63	18	54
4	22	100	26	104
5	13	55	12	60
6	5	30	6	36
7	6	42	3	21
8	2	16	3	24
9	1	1	1	9
≥ 10	2	29	2	27
Total	116	405	116	405

ECG, electrocardiographic; CMR, cardiac magnetic resonance; and LV, left ventricle

Table 2. Anthropometric heritability (QTDT)

phenotype	number of phenotyped individuals	chi-squared statistic	p-value	heritability	SE	random (individual-specific) environmental variance (Ve)	additive genetic variance (Vg)	Box-Cox transformation	covariates
BMI	433	40.55	1.9E-10	0.51	0.08	11.130	11.459	identity	.
BSA	433	42.08	8.8E-11	0.63	0.10	0.003	0.005	log	sex, age
height	433	87.84	7.1E-21	0.75	0.08	0.001	0.003	identity	sex, age
weight	433	35.5	2.6E-09	0.55	0.09	0.014	0.017	log	sex
DBP (tx adjusted)	423	3.1	0.0783	0.19	0.11	56.928	13.192	identity	sex
SBP (tx adjusted)	423	2.62	1.1E-01	0.18	0.11	0.009	0.002	log	age, weight

Box-Cox transformation: this was used to reduce heteroscedasticity and the influence of outliers where necessary - identity means no transformation was applied. Covariates: significant ($p<0.01$) covariates were selected by forward-backwards stepwise regression DBP and SBP are diastolic and systolic blood pressures adjusted for treatment (i.e. DBP + 10mmHg, SBP + 15mm Hg). Heritability was estimated by fitting variance-components models to the phenotype data by means of the QTDT computer programme (PMID: 10631157).

Table 3. ECG heritability (QTDT)

phenotype	number of phenotyped individuals	chi-squared statistic (1 d.f.)	p-value	heritability	SE	random (individual-specific) environmental variance (Ve)	additive genetic variance (Vg)	Box-Cox transformation	covariates
Cornell duration	404	11.07	0.0009	0.29	0.09	39.698	15.945	sqrt	SBP (tx adjusted), sex, age
Cornell voltage	404	7.17	0.0074	0.25	0.09	16.378	5.518	identity	age, DBP (tx adjusted)
left ventricular mass	404	43.87	3.5E-11	0.62	0.09	0.005	0.008	log	height, SBP (tx adjusted), sex, DBP (tx adjusted)
QRS voltage	405	21.12	4.3E-06	0.41	0.09	0.017	0.012	log	sex, BMI, age, SBP (tx adjusted)
QRS voltage duration	405	14.62	0.0001	0.34	0.09	99.650	50.641	sqrt	height, BMI, SBP (tx adjusted)
Sokolow-Lyon duration	403	37.38	9.7E-10	0.57	0.09	14.049	18.315	sqrt	height, BMI, SBP (tx adjusted)
Sokolow-Lyon voltage	404	14.44	0.0001	0.35	0.09	0.235	0.128	sqrt	BMI, BSA, SBP (tx adjusted), age
left ventricular mass*	362	6.88	0.0087	0.42	0.16	0.007	0.005	log	chest lateral diameter, height, SBP (tx adjusted)

Box-Cox transformation: this was used to reduce heteroscedasticity and the influence of outliers where necessary - identity means no transformation was applied. Covariates: significant ($p<0.01$) following stepwise regression model selection, hdbp_tx and hsbp_tx are diastolic and systolic blood pressures adjusted for treatment (i.e. SBP + 15mm Hg, DBP + 10mmHg). DBP and SBP are diastolic and systolic blood pressures adjusted for treatment (i.e. DBP + 10mmHg, SBP + 15mm Hg). Heritability was estimated by fitting variance-components models to the phenotype data by means of the QTDT computer programme (PMID: 10631157).

Table 4. CMR heritability (QTDT)

phenotype	number of phenotyped individuals	chi-squared statistic	p-value	heritability	SE	random (individual-specific) environmental variance (Ve)	additive genetic variance (Vg)	Box-Cox transformation	covariates
chest volume	381	16.78	4.20E-05	0.40	0.10	0.012	0.008	log	sex, weight, age, height
left ventricular end diastolic volume	405	17.15	3.45E-05	0.41	0.10	0.019	0.013	log	BSA, sex, age
left ventricular end diastolic volume index	405	18.45	1.74E-05	0.42	0.10	0.318	0.228	sqrt	sex, age
left ventricular end systolic volume	397	10.53	0.0012	0.32	0.10	0.501	0.240	sqrt	height, age, sex, BMI DBP (tx adjusted)
left ventricular end systolic volume index	397	12.56	0.0004	0.35	0.10	0.261	0.138	sqrt	age, sex, DBP (tx adjusted)
left ventricular mass	397	3.95	0.0469	0.17	0.09	0.024	0.005	log	BSA, sex, SBP (tx adjusted), age
left ventricular mass index	397	4.21	0.0402	0.17	0.08	0.024	0.005	log	sex, SBP (tx adjusted), age
right ventricular end diastolic volume	403	16.70	4.38E-05	0.33	0.08	0.024	0.012	log	height BMI age, sex
right ventricular end diastolic volume index	403	17.39	3.04E-05	0.33	0.08	0.024	0.012	log	sex, age
right ventricular end systolic volume	403	2.36	0.1245	0.14	0.09	0.852	0.134	sqrt	height, age, sex, BMI
right ventricular end systolic volume index	403	2.33	0.1269	0.13	0.09	0.450	0.070	sqrt	sex, age
right ventricular mass	403	22.55	2.05E-06	0.44	0.09	0.023	0.018	log	height, BMI sex, age
right ventricular mass index	403	22.46	2.15E-06	0.44	0.09	0.023	0.018	log	sex, age

Box-Cox transformation: this was used to reduce heteroscedasticity and the influence of outliers where necessary - identity means no transformation was applied. Significant ($p<0.01$) covariates were selected by forward-backwards stepwise regression. DBP and SBP are diastolic and systolic blood pressures adjusted for treatment (i.e. DBP + 10mmHg, SBP + 15mm Hg). Heritability was estimated by fitting variance-components models to the phenotype data by means of the QTDT computer programme (PMID: 10631157).

Table 5. Descriptive Statistics

phenotype (ECG)	sex	no. of participants	Units	mean	SD	lower quartile	median	upper quartile	minimum	maximum	transformation	covariates
left ventricular mass	female	217	g	119.57	17.07	107.73	117.45	128.56	89.58	186.42	log	height, SBP (tx adjusted), sex, DBP (tx adjusted)
left ventricular mass	male	194	g	143.95	17.65	132.32	142.36	152.61	101.61	207.21	log	height, SBP (tx adjusted), sex, DBP (tx adjusted)
QRS voltage	female	217	mV	116.93	22.31	101.40	115.50	130.40	70.55	174.00	log	sex BMI, age, SBP (tx adjusted)
QRS voltage	male	195	mV	129.27	23.10	115.20	126.70	145.70	71.00	183.70	log	sex BMI, age, SBP (tx adjusted)
QRS voltage duration	female	217	mV.ms	10216.75	2482.98	8203.80	10135.20	11812.50	5054.00	17862.30	sqrt	height, BMI, SBP (tx adjusted)
QRS voltage duration	male	195	mV.ms	11932.61	2844.56	10475.00	11457.00	13612.00	4364.00	18012.50	sqrt	height, BMI, SBP (tx adjusted)
Sokolow-Lyon duration	female	218	mV.ms	1626.50	466.83	1315.80	1607.00	1927.00	533.60	3159.00	sqrt	height, BMI, SBP (tx adjusted)
Sokolow-Lyon duration	male	193	mV.ms	1860.84	546.20	1464.00	1782.00	2247.75	424.32	3217.50	sqrt	height, BMI, SBP (tx adjusted)
Sokolow-Lyon voltage	female	219	mV	19.01	5.30	15.30	19.00	22.80	5.80	35.10	sqrt	BMI, BSA, SBP (tx adjusted), age
Sokolow-Lyon voltage	male	193	mV	21.09	6.54	16.30	20.25	24.90	6.63	39.50	sqrt	BMI, BSA, SBP (tx adjusted), age

Box-Cox transformation: this was used to reduce heteroscedasticity and the influence of outliers where necessary - identity means no transformation was applied. Significant ($p<0.01$) covariates were selected by forward-backwards stepwise regression. DBP and SBP are diastolic and systolic blood pressures adjusted for treatment (i.e. DBP + 10mmHg, SBP + 15mm Hg).

Table 6. Heritability (GCTA-GREML)

phenotype (anthropometric and blood pressure)	no. phenotyped	no. genotyped	heritability	SE	p-value
BMI	433	392	0.51	0.10	3.79E-07
height	433	392	0.84	0.08	4.18E-24
weight	433	392	0.53	0.11	5.42E-07
BSA	433	392	0.61	0.10	4.72E-09

phenotype (cardiac MR)	no. phenotyped	no. genotyped	heritability	SE	p-value
right ventricular end diastolic volume	403	367	0.33	0.11	0.0035
right ventricular end systolic volume	403	367	0.20	0.11	0.0701
right ventricular mass	403	367	0.45	0.12	0.0001
right ventricular mass index	403	367	0.45	0.12	0.0001
right ventricular end diastolic volume index	403	367	0.34	0.11	0.0026
right ventricular end systolic volume index	403	367	0.21	0.11	0.0657
left ventricular end diastolic volume	405	369	0.47	0.12	7.24E-05
left ventricular end systolic volume	397	362	matrix not invertible		
left ventricular mass	397	362	0.27	0.12	0.0230
left ventricular mass index	397	362	0.27	0.12	0.0196
left ventricular end diastolic volume index	405	369	0.47	0.12	5.91E-05
left ventricular end systolic volume index	397	362	matrix not invertible		
chest volume	381	347	0.45	0.12	0.0001
chest antero-posterior diameter	380	346	0.34	0.12	0.0051
chest lateral diameter	380	346	matrix not invertible		

phenotype (ECG)	no. phenotyped	no. genotyped	heritability	SE	p-value
left ventricular mass (ECG)	404	364	0.60	0.10	8.47E-09
Sokolow-Lyon voltage	404	364	0.40	0.11	0.0003
Sokolow-Lyon duration	403	363	0.60	0.10	8.19E-09
Cornell voltage	404	364	0.21	0.11	0.0541
Cornell duration	404	364	0.26	0.11	0.0129
QRS voltage	405	365	0.42	0.11	0.0001
QRS voltage duration	405	365	0.39	0.11	0.0005

A total of 1,230 samples were genotyped using an Illumina GWAS SNP array (503,855 SNPs). GWAS QC filters: SNP missingness per individual (mind) < 0.05; missingness per SNP (geno) < 0.05, minimum allele frequency (MAF) > 0.01 (autosomes only). These heritability estimates are based on a method published by Zaitlan et al. (Plos Genetics 2013 PMID: 23737753) that uses SNP genotype data to calculate identity-by-state (IBS) as a proxy for identity-by-descent (IBD) information, which can then be used to estimate narrow-sense heritability estimates in mixtures of closely related and unrelated individuals in an unbiased manner. The software implementation (GCTA-GREML) is detailed at http://gcta.freeforums.net/thread/241/gcta-greml-analysis-family-data.

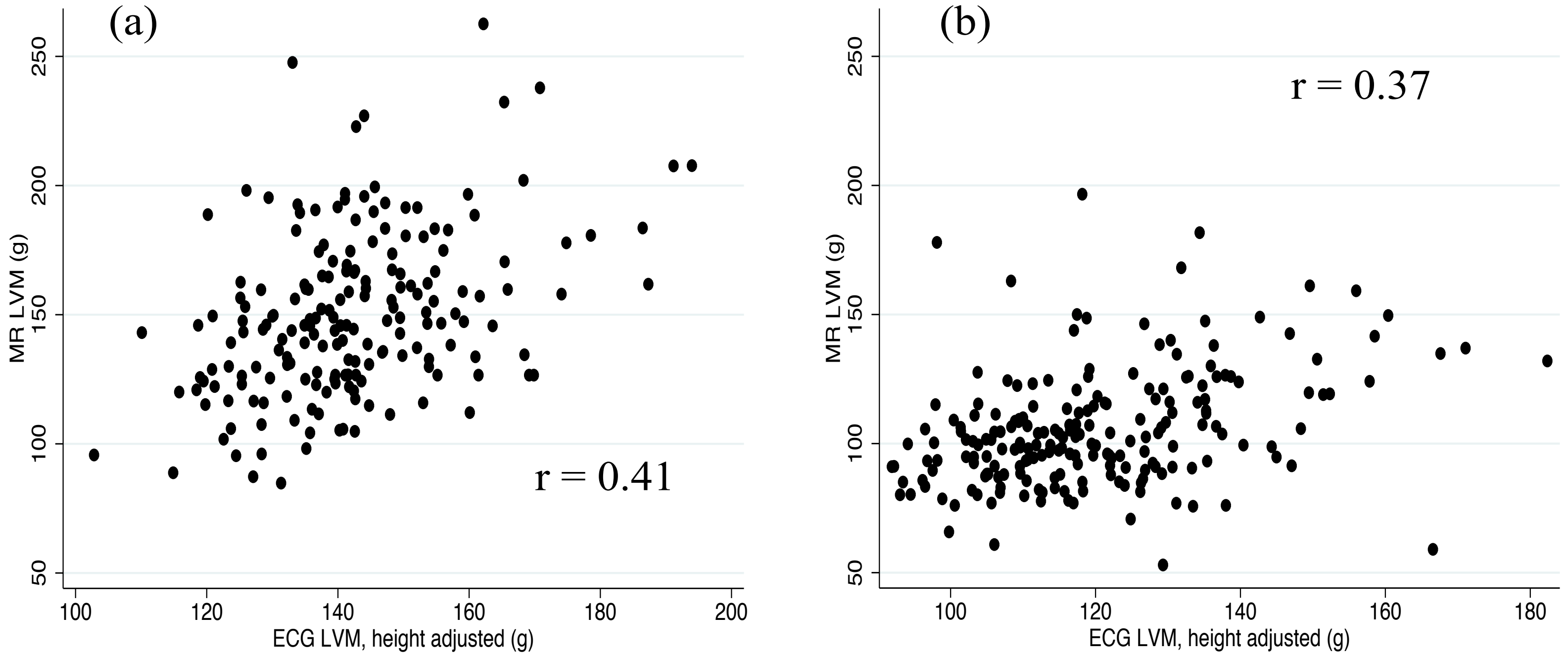

Figure S1.

The figure shows the correlation of cardiac MR and ECG measured of left ventricular (LV) mass in males (a) and females (b). ECG LVM is adjusted for height.
